# The complete chloroplast genome of *Ocimum tenuiflorum* L. subtype Krishna Tulsi and its phylogenetic analysis

**DOI:** 10.1080/23802359.2021.1951133

**Published:** 2021-07-15

**Authors:** NM Kavya, Raju Balaji, Tanuja, Madasamy Parani, Palanisamy Senthilkumar

**Affiliations:** Center for DNA Barcoding, Department of Genetic Engineering, SRM Institute of Science and Technology, Kattankulathur, Tamil Nadu, India

**Keywords:** Complete chloroplast genome, Lamiaceae, *Ocimum tenuiflorum*, Krishna Tulsi, phylogenetic analysis

## Abstract

The complete chloroplast genome (cp) of *Ocimum tenuiflorum* L. subtype Krishna Tulsi was sequenced and assembled using Illumina paired-end sequencing data. The cp genome is 151,758 bp in length, including a large single copy (LSC) region of 82,794 bp, a small single-copy region (SSC) of 17,592 bp, and a pair of inverted repeated (IR) region of 25,686 bp. The cp genome of Krishna Tulsi encodes 129 genes, including 90 protein-coding, 31 transfer RNA (tRNA), and eight ribosomal RNA (rRNA) genes. While the overall GC content was 37.9%, it is 36.0%, 31.8%, and 43.1% in the LSC, SSC, and IR regions, respectively. Phylogenetic analysis based on chloroplast genome sequences of 17 species from Lamiaceae revealed that *O. tenuiflorum* subtype Krishna Tulsi is clustered with other *Ocimum* species, and forms a clade with genera from family Lamiaceae.

*Ocimum* is a genus of aromatic perennial herb in the family Lamiaceae and includes more than 150 species widely distributed in paleotropics (Bast et al. 2014). It is known as ‘Queen of Herbs’ within Ayurveda because of its enormous therapeutic applications (Cohen 2014). *Ocimum tenuiflorum* L. (commonly known as Holy Basil) possesses antimicrobial, antioxidant, anticancer, antidiabetic, and cardioprotective properties conferred by its bioactive compounds (Baliga et al. 2013). *Ocimum tenuiflorum* has two subtypes, Krishna Tulsi with purple leaves and Rama Tulsi with green leaves. The leaves and roots of Krishna Tulsi are used for treating bronchitis, skin diseases, fever, asthma, and arthritis (Verma 2016). In this study, we assembled and annotated the complete chloroplast genome of Krishna Tulsi (GenBank accession number: MW724787) to provide genetic resources for further research.

Plant material of *O. tenuiflorum* subtype Krishna Tulsi was collected from Thailavaram, Chengalpattu district, Tamil Nadu, India (GPS coordinates: 12° 49′ 44.5ʺ N 80° 02′ 43.9ʺ E). Voucher specimen was deposited at the SRM Institute of Science and Technology Herbarium (http://www.srmist.edu.in/, Dr. P. Senthilkumar, senthilp3@srmist.edu.in) under the voucher number SRMH000144. Total genomic DNA was extracted from fresh leaves as described before (Nithaniyal et al. 2017) and the genomic DNA was stored at −80 ºC until use. A whole-genome DNA sequencing library was constructed using the Nextera XT Library Prep Kit (Illumina, CA, USA). The DNA library (2 × 150 bp) was sequenced on NovoSeq 6000 platform (Illumina, CA, USA) and obtained 1.584 Gb of 2 × 150 bp paired-end sequencing data. Approximately 1.574 Gb of Q20 data (99.3%) obtained after adaptor removal and quality filtering using FastQC (Wingett and Andrews 2018) and Trimmomatic v0.39 (Bolger et al. 2014). The chloroplast genome of Krishna Tulsi was assembled using NovoPlasty v.4.3.1 (k-mer 33) (Dierckxsens et al. 2017) with *O. tenuiflorum* (NC043873) as a reference seed sequence. The minimum, maximum and mean depth of coverage of the assembled genome was 16X, 3960X, and 1252X, respectively. The annotation of the assembled Krishna Tulsi chloroplast genome was performed with GeSeq (Tillich et al. 2017) using the chloroplast genomes of *O. basilicum* (NC035143.1) and *O. tenuiflorum* (NC043873.1) as reference sequences. The predicted tRNAs were annotated by tRNAscan-SE 2.0 (Lowe and Chan 2016).

The complete chloroplast genome of *O. tenuiflorum* subtype Krishna Tulsi (GenBank accession: MW724787) was 151,758 bp in length, including a large single-copy (LSC) region of 82,794 bp, a small single-copy (SSC) region of 17,529 bp, and a pair of inverted repeated (IR) region of 25,686 bp. The complete chloroplast genome contained 129 genes, including 90 protein-coding genes, 31 transfer RNA (tRNA) genes, and eight ribosomal RNA (rRNA) genes. The GC content of the complete chloroplast genome was 37.9%, and the corresponding values of the LSC, SSC, and IR were 36.0%, 31.8%, and 43.1%, respectively. Introns were present in 10 annotated genes. Among them, six protein-coding genes (atpF, rpoC1, rps12, rpl2, ndhA, ndhB) had a single intron and two protein-coding genes (ycf3 and clpP1) had two introns. Besides, two tRNA genes (trnI-GAU and trnA-UGC) had single intron. We also identified two microsatellites (SSRs), (GA)6 and (TA)6, in the chloroplast genome using MISA (Beier et al. 2017).

A maximum-likelihood tree of molecular distance analysis was performed using 1000 bootstrap replicates with the Jukes-Cantor model in MEGA version X (Kumar et al. 2018) from the alignments generated in MAFFT (Katoh and Standley 2013). *Verbena officinalis* L. (Verbenaceae) and *Lancea hirsuta* Bonati. (Phrymaceae) were designated as outgroups, and 17 published complete chloroplast genome sequences from the Lamiaceae family were included as in group taxa. The phylogenetic analysis of *O. tenuiflorum* subtype Krishna Tulsi revealed that it is closely related to *O. tenuiflorum*, *O. gratissimum,* and *O. basilicum* (Figure 1). This study could lay a foundation for species delimitation, phylogenetic analysis, DNA barcoding, and evolutionary relationships among and within genus *Ocimum*.

**Figure 1.**
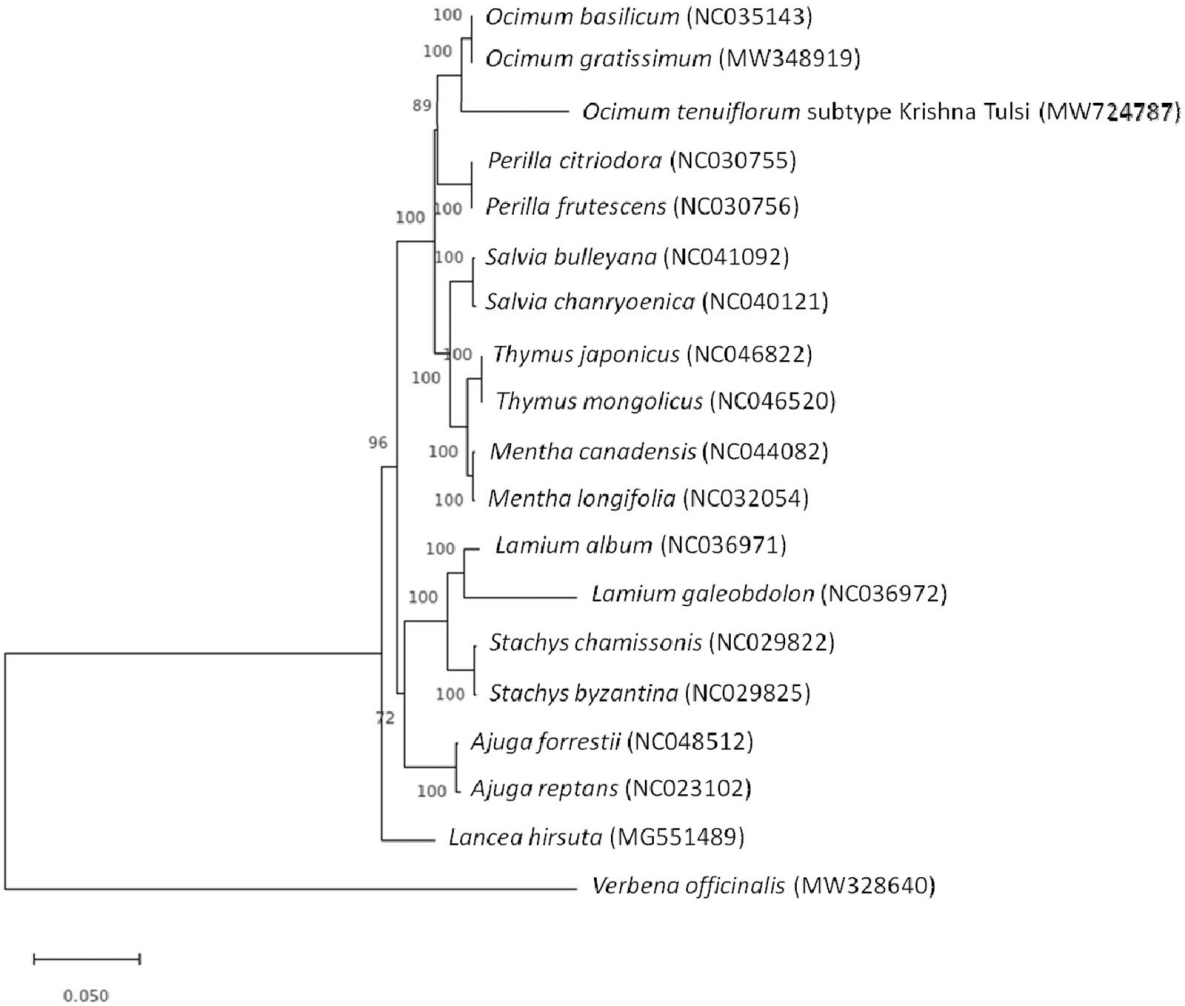
Maximum-likelihood tree based on the whole chloroplast genome sequences of 19 species, including *Verbena officinalis* L. and *Lancea hirsuta* Bonati, as outgroups. The bootstrap support values >50% from 1000 replicates are given at the nodes.

## Data Availability

The data that support the findings of this study are openly available in NCBI at https://www.ncbi.nlm.nih.gov/nuccore/MW724787.1/, under GenBank accession number MW724787. The NGS sequencing data files are available from the BioProject, SRA, and Bio-Sample ID under the accession numbers PRJNA706881, SRR13862647, and SAMN18147081, respectively.
